# A Comparative Study of Cr(VI) Sorption by *Aureobasidium pullulans* AKW Biomass and Its Extracellular Melanin: Complementary Modeling with Equilibrium Isotherms, Kinetic Studies, and Decision Tree Modeling

**DOI:** 10.3390/polym15183754

**Published:** 2023-09-14

**Authors:** Hala Fakhry, Abeer A. Ghoniem, Fatimah O. Al-Otibi, Yosra A. Helmy, Mohammed S. El Hersh, Khaled M. Elattar, WesamEldin I. A. Saber, Ashraf Elsayed

**Affiliations:** 1 National Institute of Oceanography and Fisheries (NIOF), Cairo 11865, Egypt; 2 Department of Aquatic Environmental Science, Faculty of Fish Resources, Suez University, Suez 43518, Egypt; 3 Microbial Activity Unit, Department of Microbiology, Soils, Water and Environment Research Institute, Agricultural Research Center, Giza 12619, Egypt; abeer.abdelkhalik@yahoo.com (A.A.G.); m.elhersh@yahoo.com (M.S.E.H.); 4 Botany and Microbiology Department, Faculty of Science, King Saud University, Riyadh 11451, Saudi Arabia; falotibi@ksu.edu.sa; 5 Department of Veterinary Science, Martin-Gatton College of Agriculture, Food, and Environment, University of Kentucky, Lexington, KY 40546, USA; yosra.helmy@uky.edu; 6 Unit of Genetic Engineering and Biotechnology, Faculty of Science, Mansoura University, Mansoura 35516, Egypt; khaledelattar2@yahoo.com; 7 Botany Department, Faculty of Science, Mansoura University, Mansoura 35516, Egypt; ashraf-badawy@mans.edu.eg

**Keywords:** artificial intelligence, biosorption, fungal biomass, water treatment, heavy metals

## Abstract

Melanin as a natural polymer is found in all living organisms, and plays an important role in protecting the body from harmful UV rays from the sun. The efficiency of fungal biomass (*Aureobasidium pullulans*) and its extracellular melanin as Cr(VI) biosorbents was comparatively considered. The efficiency of Cr(VI) biosorption by the two sorbents used was augmented up to 240 min. The maximum sorption capacities were 485.747 (fungus biomass) and 595.974 (melanin) mg/g. The practical data were merely fitted to both Langmuir and Freundlich isotherms. The kinetics of the biosorption process obeyed the pseudo-first-order. Melanin was superior in Cr(VI) sorption than fungal biomass. Furthermore, four independent variables (contact time, initial concentration of Cr(VI), biosorbent dosage, and pH,) were modeled by the two decision trees (DTs). Conversely, to equilibrium isotherms and kinetic studies, DT of fungal biomass had lower errors compared to DT of melanin. Lately, the DTs improved the efficacy of the Cr(VI) removal process, thus introducing complementary and alternative solutions to equilibrium isotherms and kinetic studies. The Cr(VI) biosorption onto the biosorbents was confirmed and elucidated through FTIR, SEM, and EDX investigations. Conclusively, this is the first report study attaining the biosorption of Cr(VI) by biomass of *A*. *pullulans* and its extracellular melanin among equilibrium isotherms, kinetic study, and algorithmic decision tree modeling.

## 1. Introduction

Admission to hygienic and secure water is crucial for healthy living organisms; however, this reality could not be achieved worldwide owing to the increase in human activity e.g., agricultural practices, and industrialization [1]. Heavy metals are a major environmental anxiety owing to their toxicity, ability to bioaccumulate, and persistence in the food series [2]. Chromium is produced in the environment as a byproduct of several activities such as mining, energy production, steel, and iron industries. However, chromium ions at permissible limits could possess a vital function in the biosynthesis of nucleic acids, and the metabolism of fat, proteins, and carbohydrates. The toxicity of Cr ions was found to arise from oxidation status [3]. The high oxidative form (hexavalent Cr^6+^) is more toxigenic, and carcinogenic in biological systems compared to the trivalent Cr^3+^ ions, which are less toxic, more soluble, and have stable oxidation status [4]. 

A variety of water bioremediation processes (e.g., ion exchange, and coagulation-flocculation), are utilized to remove heavy metals. However, these procedures have many disadvantages i.e., expensive, energy-intensive requirements, time-consuming, and undergo drawbacks correlated to recyclability. Therefore, the biotechnological-based treatments, e.g., bacteria, fungi, plants, and algae, are, currently, being documented on leaching heavy metals from contaminated effluents [5,6], since the biological methods are cost-effective and eco-friendly [7].

Melanin is a black or dark brown high molecular polymer, which is biosynthesized by oxidation and polymerization of phenolic compounds [8]. It is associated with some melanized fungal strains, situated in the cell wall or remains extracellularly [9]. Melanin also has many other biological properties, including antimicrobial [10], photoprotective [11], and antioxidant effects. Melanin has been shown to inhibit the growth of bacteria, fungi, and viruses. This is thought to be due to their ability to chelate metal ions and disrupt the cell membranes of microorganisms [12]. Melanin absorbs ultraviolet radiation, which can damage DNA leading to skin cancer [13]. They also help to prevent premature aging of the skin [14].

Both melanized fungi and their melanin are new renewable potential biosorbents for removing heavy metals [15]. Fungal melanin contains a diversity of functional groups, including phenolic, carboxylic, amine, and hydroxyl groups that can bind to metal ions [16]. For instance, copper binds mainly to the carboxyl group in catechol melanin at pH ˂ 5, but at pH ˃ 6, copper binds primarily to the phenolic hydroxyl group [17]. Another study reported that the proteinaceous fraction containing nitrogen groups can bind iron and copper [18].

Additionally, the biosorbent potential of melanin depends upon the physicochemical features of functional groups [19,20]. Moreover, melanin polymers could also interact with metal ions through their free radical species, forming complexation [21,22]. Another, the thermal tolerance of melanin and insolubility in organic solvents and aqueous solutions ensure the constancy of melanin as an adsorbent to industrial effluents containing metals [23]. 

Numerous reports discussed the ability of filamentous fungi to bind metals, where fungal mycelium involves an interconnected network of millimeter-to-centimeter-long hyphae [24]. Moreover, fungal biomass can produce massive growth on a large scale, where the biosorption of metal ions by filamentous fungi can occur at a minimal cost [25].

Decision trees (DTs) are a kind of artificial intelligence classified as supervised machine learning and data mining algorithms. DTs are used to solve both classification and regression tasks, determine the relative importance of the independent variable on a response target, clustering analysis, and understand and interpret a wide variety of data types. DTs work by recursively partitioning a data set into minor subsets until each subset is homogeneous concerning the target variable. The decision rules at each node of the tree are learned from the training data and can be used to predict the target variable for new data points [26]. To obtain optimum prediction by DT for new conditions, it is crucial to have an applicable assortment of data. Anyhow, the training and optimization process should be followed by authentication, as the certainty tree can be influenced by overfitting and other affairs thus several roles are recommended to avoid such problems [9,27]. 

No information is available about the combination of DT with equilibrium isotherms and kinetic studies for the determination of relative variable importance and modeling the biosorption process. Combining equilibrium isotherms and kinetic studies with DTs can provide a holistic understanding of the sorption process. Where equilibrium isotherms describe the system relationship between the amount of solute adsorbed onto a sorbent, kinetics focus on how quickly the sorption process occurs and how the concentration of the solute changes over time, DT, on the other side, can predict the outcome or behavior of all sorption system based on various input parameters, as well as determining the relative importance of the independent variables. As a case study, *Aureobasidium pullulans* and its extracellular melanin were used during the current work in a trial to understand the mechanism by which pollutants are adsorbed onto fungal biomass and melanin. In general, the exhaustion of fungal biomass or melanin in the eradication of heavy metal ions from wastewater is an urgent demand from the environmental arguments of view.

Biomass-based sorbents are a sustainable and cost-effective way to remove Cr(VI) from water and wastewater [28]. These sorbents can be made from a variety of biomass materials, such as agricultural waste, wood waste, and food waste. They can be functionalized with various chemical groups, such as carboxylates, amines, and phosphates, to improve their Cr(VI) sorption capacity. The sorption of Cr(VI) onto biomass-based sorbents is a complex process that is influenced by several factors, including the sorbent material, the functionalization chemistry, the Cr(VI) concentration, the pH, and the temperature. In general, Cr(VI) sorption is favored by low pH values and high temperatures [29]. Biomass-based sorbents are effective in removing Cr(VI) from water and wastewater with high sorption capacities and low regeneration costs [30]. They are promising technologies for the removal of this toxic heavy metal from the environment. The benefits of using functionalized biomass-based sorbents for Cr(VI) removal included [31]: (1) They are renewable and biodegradable, making them ecofriendly. (2) They are low-cost and easy to produce. (3) They can be tailored to specific Cr(VI) removal applications. Functionalized biomass-based sorbents are a promising new technology for the removal of Cr(VI) from water and wastewater. They offer many advantages over traditional sorbents, making them a more sustainable and cost-effective option for environmental remediation [32].

Herein, our investigated study focused on Cr(VI) biosorption through (i) comparing the biomass of *A. pullulans* and its extracellular melanin during isotherm models i.e., Langmuir, Freundlich, and Temkin, (ii) investigating the kinetic behavior by applying pseudo-first-order and pseudo-second-order (iii) modeling the overall biosorption process by DT for both fungal biomass and melanin. Additionally, it confirmed the biosorption process of Cr(VI) ions on fungal biomass and melanin particles by SEM, EDX, and FT-IR analyses. The current study provides complementary information on modeling biosorption using different modeling procedures. Moreover, developing efficient and environmentally friendly methods for heavy metals removal from industrial wastewater. Cr(VI) was chosen as a case study. As fungal biomass and melanin are renewable, and ecofriendly resources that are readily available, it is important to compare the sorption capacity of both biomaterials using two complementary models to determine the most efficient biosorption process for a particular application.

## 2. Materials and Methods

### 2.1. Materials

All the used chemicals in the current study were purchased from Sigma Co., St. Louis, USA. HCl (0.1 N), and NaOH (0.1 N) solutions were prepared and utilized to adjust the pH of the solution.

### 2.2. Preparation of Hexavalent Chromium Solution

Potassium dichromate (Cr(VI), ≥99%) solution was prepared at 1000 mg/L in deionized water and used to prepare a concentration range of 5 to 200 mg/L. The working solution pH was adjusted to the respective level by HCl or NaOH using a pH meter (HI98130, Hanna Instruments, Romania). Trials were performed in a 100 mL working solution in a 250 mL conical flask. The remaining Cr(VI) after the biosorption process was determined [33] using an Atomic Absorption Spectrophotometer (Buck Scientific Accusys 211 series, Norwalk, Connecticut, USA) by an air/acetylene flame system.

### 2.3. Preparation of Biosorbants

#### 2.3.1. Fungus and Melanin

*Aureobasidium pullulans* AKW (GenBank accession number OP924554) was obtained in an earlier study [9]. The medium of melanin biosynthesis [34] consisted of potato sucrose broth. The medium was prepared by mixing potato infusion (200 g of sliced, and unpeeled potatoes were boiled in 1 L of distilled water for 30 min, then filtered to remove debris of potato) and sucrose (50 g/L of potato infusion). The medium was adjusted to pH 6.0. Flasks containing 100 sterilized medium (15 min at 121 °C) were inoculated by fungal seeds (3 × 10^7^ cells/mL) in a proportion of 5%. The culture was incubated at 30 °C for 10 days with a rotation speed of 200 rpm. 

#### 2.3.2. Separation and Purification of Melanin

The separation and purification of melanin was carried out following The protocol of El-Gamal et al. [35] and Müjdeci [36] with minor modifications. Briefly, melanin was separated from the microbial cell pellets by centrifugation (15 min, 3000× *g*). Melanin in the cell-free filtrate was precipitated by reducing the pH down to 2.0 by HCl (6 M) and kept for 4 h under cooling. The precipitate was separated at 7000× *g* for 15 min. The recovered melanin was washed with distilled water. The melanin purification process was repeated four times followed by lyophilization, then stored (at −20 °C).

### 2.4. Cr(VI) Ions Biosorption Process

The batch adsorption unit contained 0.01 g of the dried fungal biomass or the biosynthesized melanin and 10 mg/L Cr(VI). 100 mL solution was then agitated (200 rpm at room temperature) in an orbital shaker. Solutions were gathered at a definite time and then centrifuged (12,000 rpm, 10 min). Cr(VI) residual concentrations were analyzed using ICP-OES [37] and the amount of metal ions biosorbed onto the used biosorbents was estimated (Equation (1)).

(1) $$q_{e}=\frac{\left({C\;}_{0}-{C\;}_{e}\right)v}{w}\;$$

where q_e_ is the quantity of Cr(VI) ions absorbed at a specific period per unit mass of the used materials (mg/g), C_0_ is the initial Cr(VI) level (mg/L), C_e_ is the residual Cr(VI) in solution at equilibrium (mg L^−1^), V is the volume (L) and W is the amount of the used biosorbent particles (g). The Cr(VI) ions removal percentage was estimated by Equation (2):

(2) $$\%\;\mathrm{of}\;\mathrm{Cr}\left(\mathrm{VI}\right)\;\mathrm{ions}\;\mathrm{removal}=\frac{C_{0}-C_{e}}{C_{0}}\times 100$$

where C_o_ and C_e_ (mg/L) are the initial and equilibrium levels of Cr(VI) ions in the solution.

The impacts of numerous processing factors, i.e., initial Cr(VI) ions concentration, interaction time, solution pH, and weight of biosorbed material on the Cr(VI) biosorption process were optimized and studied.

#### 2.4.1. Influence of Contact Time

A weighing 0.01 g of the fungal biomass or the biosynthesized melanin particles was added to a 100 mL volume of Cr(VI) solution (10 ppm). Then, the solutions were shaken for diverse intervals of time (10, 20, 30, 60, 120, 180, 240, 280, and 300 min).

#### 2.4.2. Effect of Initial Cr(VI) Concentration

A weight of 0.01 g of the used fungal biomass or the biosynthesized melanin particles was added to a series of initial Cr(VI) solution concentrations (5, 10, 25, 50, 100, and 200 mg/L), and the tests were accomplished for the equilibrium.

#### 2.4.3. Effect of Thresholds of Fungal Biomass and Its Melanin

Different amounts of the used fungal biomass and the biosynthesized melanin particles (0.005, 0.01, 0.05, 0.1, and 0.2 g) were added to each 100 mL of Cr(VI) aqueous solution and shaken for a constant biosorption time.

#### 2.4.4. Solution pH vis. Cr(VI) Sorbtion

The pH effect of the Cr(VI) solution was inspected at diverse pH values (2, 4, 5, 7, 9, and 11) using a pH meter (ADWA, AD1000, Romania), and it was controlled by adding (1 N) NaOH or HCl solutions. The bio-sorbent particles (0.01 g) were added to each 100 mL of Cr(VI) solution using 10 ppm as an initial concentration and were shaken for a constant bio-sorption time. 

### 2.5. Biosorption Isotherm Determination

To study the biosorption isotherms (equilibrium modeling), the impact of the initial Cr(VI) ions level on the biosorption practicability was conducted through batch mode studies by contacting 0.01 g of the used bio-sorbents within a range from 5 to 200 mg/L Cr(VI).

Equilibrium isotherm models defined the adsorbent and adsorbate interactions. The shape of isotherms is determined by many factors such as Cr(VI) level in the solution, their biosorbent capabilities, and the grade of rivalry between Cr(VI) ions to bind to the active sites of biosorbent. The biosorption behavior of Cr(VI) ions onto the used bio-sorbent particles and the optimal terms of biosorption attitude can be appraised from the equilibrium isothermal factors. The Langmuir, Freundlich, and Temkin adsorption isotherm models were applied to explain the equilibrium discrimination of Cr(VI) ions onto the biosorbents [38]. Langmuir isotherm assumes the homogeneity of the biosorption process, for instance, biosorption uniform energy with no transmigration of Cr(VI) ions in the plane of the used bio-sorbent particles surface, with a monolayer coverage, and uniform biosorption energies. The linear isotherm form of Langmuir is presented in Table 1. 

The model of Freundlich biosorption isotherm suggested that the Cr(VI) ions uptake takes place on a heterogeneous surface by multilayer biosorption with the lateral interaction between biosorbent Cr(VI) ions on the surface of used biosorbent particles surface. The linear form of Freundlich isotherm is publicized in Table 1. The Temkin isotherm transacts with the indirect impacts of interactions between the biosorbent and biosorbent Table 1. The heat of biosorption of all molecule layers decreases linearly with coverage owing to the interactions between bio-sorbent, and biosorbate [40]. 

### 2.6. Biosorption Kinetic Studies

The biosorption kinetic study is a crucial step in highlighting the biosorption process’s mechanism. An amount of 0.01 g of the used fungal biomass and the biosynthesized melanin particles were mixed with 10 ppm Cr(VI) metal ions at 200 rpm for diverse intervals of time (10–300 min). The final Cr(VI) concentration was measured after each interval time and the amount of biosorbent metal ion (q_t_, mg/g) was plotted versus time (t, min) for kinetic modeling. Four prevalent kinetic models (pseudo-first-order, pseudo-second-order, intra-particle Diffusion, and Elovich) were searched to find out the biosorption of Cr(VI) onto the used biosorbent particles. Lagergren’s pseudo-first-order supposes that the biosorption process relies merely upon the level of Cr(VI) ions in the aqueous phase and the biosorbent’s accessible binding sites at any time [41]. The pseudo-first-order model Equation is presented in Table 2.

The pseudo-second-order kinetic model proposed that chemical biosorption is the rate-controlling step of a biosorption process (Table 2). An intra-particle diffusion model is a mechanistic sequence used to clarify biosorption kinetics. Generally, the biosorption process is controlled by either external, pores, or surface diffusion adsorption on the surface of the pore or a mixture of them. It is a function of adsorption aptitude and time that is calculated by the Equation given in Table 2 as reported by Weber and Morris [42].

**Table 2:** Pseudo-first-order, Pseudo-second-order, Elovich, and Intra-particle diffusion kinetics models equations for biosorption of Cr(VI) ions onto the used fungal biomass and the biosynthesized melanin at 25 °C [38,43].

Adsorption Kinetic Models	Equation	Parameter
Pseudo-first-order	$ln\;\left(q_{e}-q_{t}\right)=\ln q_{e}-K_{1}t\;\;\;$	q_t_ and q_e_ are the biosorbent Cr(VI) ions amount at time t and equilibrium (mg/g), respectively. k_1_ (min^−1^) is the first-order reaction rate constant
Pseudo-second-order	$\frac{t\;}{q_{t}}=\frac{1}{\;{\left(K_{2}q_{e}\right)}^{2}}$	qt and q_e_ are the biosorbent Cr(VI) ions amount at time t and equilibrium (mg/g), respectively, and k_2_ is the second-order reaction rate equilibrium constant (g/mg/min).
Elovich	*q_t_ = ὰ +ß ln t*	ὰ is the initial sorption rate (mg/g/min) and ß is the extent of surface coverage and activation energy for chemisorption (g/mg)
Intra-particle diffusion	$q_{t}=k_{i}t^{0.5}+c_{i}$	k_i_ is the intra-particle diffusion rate constant, and c_i_ gives a prediction about the boundary layer thickness

### 2.7. Decision Tree Learning Algorithm

Two parametric machine learning regression and classification trees were constructed [9] to find out the probable answers for maximizing Cr(VI) removal using the 4 tested variables that were used during equilibrium isotherms and kinetic studies, i.e., contact time, initial Cr(VI) concentration, pH, and fungal biomass or melanin doses. The relative importance of the 4 input continuous predictor variables that mitigate the noise resulting from the experiential procedures was tested. The target response variable was Cr(VI) removal. The 4 continuous predictors in the data were used to generate DT based on the least squared errors rule as a node-splitting process.

The DT statistics for training and testing to select and evaluate the optimal tree, where the maximum correlation coefficients (R^2^) and lowest model error (root mean squared error (RMSE), mean squared error (MSE), mean absolute deviation (MAD), and mean percent error (MAPE), and standard deviation, were used judgment statistics.

For both fungal biomass and melanin, validation was carried out at a random fraction of 0.3, where 30% of data were used for testing and 70% for training. A total of 75 data values, representing three repeated experiments of the equilibrium isotherms and kinetic studies, were used. This procedure was performed to evaluate the prediction precision and avoid overfitting.

During the learning process of DT, the nodes continuously split till the terminal nodes could not be further split. Although deeper trees have more precise predictions, the deepness of the DT was stopped when further modifications did not decrease MSE.

#### 2.7.1. Model Validation of DTs

The predicted conditions at various nodes for both DT models were experimentally validated using three different combinations of the 4 parameters. The predicted and observed values were emulated to judge the model’s precision in maximizing Cr(VI) removal.

#### 2.7.2. Software and Statistical Procedure

The data retrieved from the equilibrium isotherms and kinetic data were used as historical data for machine learning. Experiments of Cr(VI) removal percentage by fungal biomass or melanin were repeated thrice, and presented as the average with standard deviation (±SD). The algorithm of DT and the statistical calculations were implemented using Minitab software (version 21, Minitab Inc., State College, PA, USA).

### 2.8. Characterization of the Biosorption Process

Information about the surface morphology and topography of the used fungal biomass and its extracellular melanin particles, as well as their surface chemical composition, were established [44] by scanning electron microscopy (SEM), and energy dispersive x-ray (EDX) on JSM6380, JEOL, Japan. The samples were covered with a thin layer of gold to avoid charging during imaging.

The basic functional groups, and surface chemistry of the fungal biomass, and melanin were scrutinized by Fourier-transform infrared spectroscopy (FT-IR), in which the spectral analysis was run at Thermo-Fisher Nicolet IS10, USA Spectrophotometer at a resolution of four cm^−1^ in the wavenumber ranges of 500–4000 cm^−1^ in transmission approach to identify the influence of the functional groups in the adsorption of Cr(VI) ions on the surface of fungi and melanin. In this route, 20 mL of 1000 mg/L of Cr(VI) solution was mixed with 0.1 g of each solid melanin and fungi biomass at 45 °C. Centrifugation at 200 rpm of the solution was achieved after incubation for 24 h to separate the melanin particles and powdered fungi. The separated pellets were washed with ethanol and dried in air. The solid melanin and fungi biomass were immersed in distilled water, separated following the matching aforementioned conditions, and used as a control.

## 3. Results and Discussion

**Figure 1:**
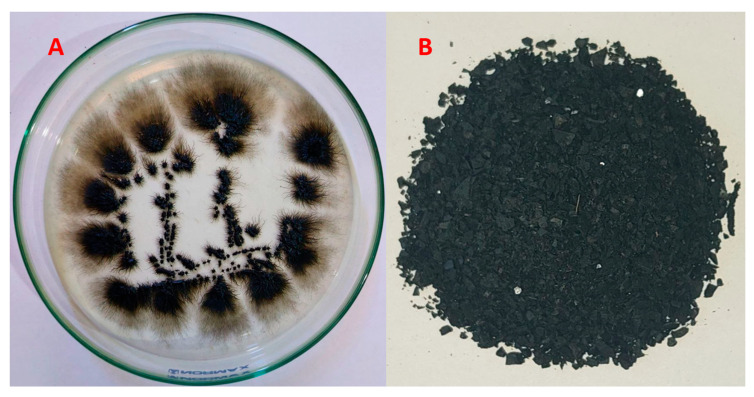
A close-up view of the *A. pullulans* biomass on growth medium (**A**), as well as the biosynthesized melanin by the fungus (**B**).

Melanin was produced extracellularly by *A. pullulans*. The resulting melanin after fermentation was harvested, purified, dried, and ground before being used in the biosorption process (Figure 1).

The technological significance of heavy metals has been recognized, since the outbreaks of metals in a lot of industrial processes, such as electrical equipment, paints, treated wood, and lead-acid batteries. Subsequently, a massive quantity of heavy metals has been discharged into the ecosystem [45]. As the metals are discharged at doses beyond accepted concentration, human life is threatened [46].

The biosorption process has several advantages compared to other ones e.g., less sludge, cheap running, and installation cost, and flexible design process [46,47]. Likewise, fungal cells play a major role in the biosorption process of heavy metals, because of their content of functional groups that attract and sequester metals on their surface [5]. Wherein, the fungal biomass of *A*. *pullulans* and its extracellular melanin were comparatively studied as biosorbent agents for Cr(VI) eradication from aqueous solution.

### 3.1. Effect of Contact Time

**Figure 2:**
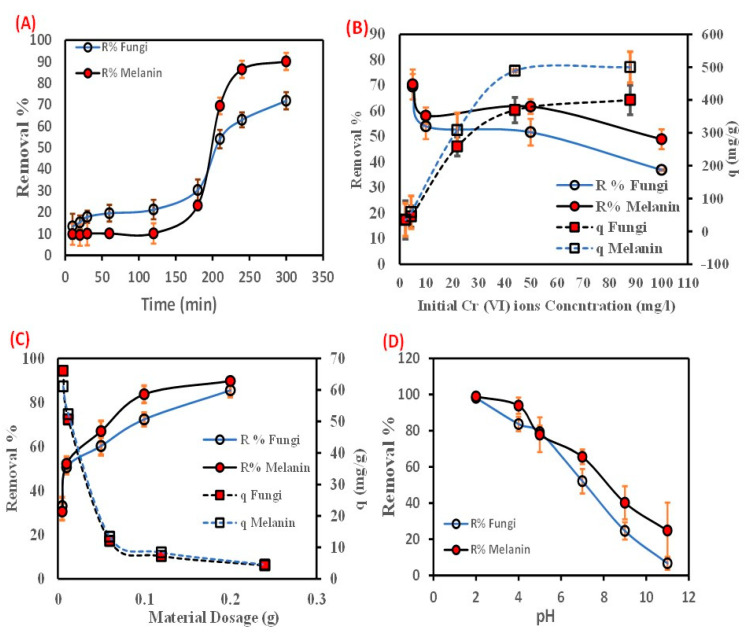
Effect of batch biosorption parameters (**A**) contact time (min), (**B**) initial Cr(VI) metal ions concentration (ppm), (**C**) material dosage (g), and (**D**) pH on the Cr(VI) biosorption onto the used fungal biomass or the biosynthesized melanin.

The estimation and design of a biosorbent for the adsorption of Cr(VI) from an aqueous solution depend heavily on the reduction rate. The impact of contact time between Cr(VI) ions, and fungal biomass, or extracellular melanin particles was investigated during reaction intervals up to 300 min) (Figure 2A). The figured data showed that the removal percentage (R%) of Cr(VI) ions increased instantaneously with the augmenting of contact time in the first few minutes, then a slow rate of biosorption was perceived with no notable biosorption rate beyond 240 min (equilibrium time). The biosorption of Cr(VI) ions (R%) increased from 13.46 up to 71.80% and from 9.83 up to 90.06% with increasing the contact time from 10–300 min, by fungal biomass and its extracellular melanin particles, respectively. The biosorption process of Cr(VI) ions is accomplished in two steps. The first step was a fast approach that persisted for a short time. The second stage involved a more gradual procedure that persisted until equilibrium was established. Further increases in contact time after the equilibrium time did not result in any additional removal percentage. The difficulties that Cr(VI) ions encounter in occupying the residual free active surface sites might be the cause of the reduced biosorption rate in the later stage. This is attributed to the forces between the Cr(VI) ions in the solid and bulk phases. The decline in Cr(VI) ions R% with increasing contact time could be a result of the biosorption progression being dominated by the intraparticle diffusion process [48].

Furthermore, metal biosorption is a biphasic process that involves the prompt metal ions sorption on the active groups located on the surface of biomass in the first phase, accompanied by the diffusion of metal throughout the biomass’ interior binding sites in the second phase [49]. Moreover, biosynthesized melanin was found to have a higher affinity for adsorbing Cr(VI) than the native fungal biomass. The difference in removal percentage between the two was up to 10%. The literature has reported a similar tendency of chromium adsorption utilizing diverse biomasses as a function of contact time, for example, *Spirulina* sp. biomass [50], *Chlorella* species biomass [51], and dead fungal biomass of *Phanerochaete chrysosporium* [52].

### 3.2. Effect of Initial Concentration

The impact of the initial concentration of Cr(VI) ions on its R % by the fungal biomass and its extracellular melanin was investigated (Figure 2B). As depicted by the data, there is a reduction in R (%) of Cr(VI) ions correlated with increasing initial concentration and vice versa with the biosorption aptitude. The increase in the concentration of Cr(VI) ions from 5 up to 200 ppm led to a notable decrease in the R (%) of Cr(VI) ions from 69.493 to 20.000%, and 70.373 to 25.000%, by fungal biomass and its extracellular melanin, respectively.

While the biosorption aptitude of Cr(VI) ions increased from 34.747 to 400.000 mg/g, and from 35.187 to 500.000 mg/g onto fungal biomass and extracellular melanin, respectively. This phenomenon might be due to the fast depletion of the biosorption sites [53]. Moreover, the increase in the biosorption aptitude might be ascribed to the presence of Cr(VI) molecules in the aqueous phase, which causes the rapid movement of the molecules to the used bio-sorbent particle surface at higher concentrations, leading to an elevation in the coefficient of mass transfer [54]. This result is consistent with other reported studies [55]. The results signify that the biosynthesized melanin has a great potential for reducing Cr(VI) at higher initial concentrations than the used fungal biomass (~100 mg/g).

Concerning the obtained results, it could be deduced that the used fungal biomass and the extracellular melanin could eliminate a higher Cr(VI) amount per gram of biosorbent.

### 3.3. Dosage of Biosobants Virsus Cr(VI) Ions Biosorption

The biosorption of Cr(VI) ions was profoundly influenced by the biomass dosage during the process. Figure 2C depicted that the removal percentage (R%) of Cr(VI) ions increased with increasing the biosorbents’ dosage. However, the biosorption talent (q) decreased with increasing the biosorbents’ dosage. The removal percentage of Cr(VI) ions increased from 33.033% to 85.557% and from 30.550% to 89.763% when the dosage of the fungal biomass and the extracellular melanin increased from 0.005 g to 0.2 g, respectively. However, the biosorption capability decreased from 66.067 mg/g to 4.278 mg/g and from 61.100 mg/g to 4.488 mg/g, respectively, with increasing dosages of fungal biomass and extracellular melanin. Thus, the accessible increase in the active sites on the surface of the biosorbent is a result of the increased amount of the biosorbent in the aqueous phase [56].

Accordingly, a direct relationship between the maximum concentration of the biosorbent capacity and the loaded biomass, for instance, the lowest amount of the used biosorbent particles was already higher than the maximum amount. This inverse relationship is attributed to the agglomeration or coagulation of biomass particles at high biomass loadings. This aggregation of particles reduces the number of accessible sites for metal ion biosorption which could be attributed to the decrease in the metal-to-biosorbent ratio as the dosage was increased [57]. Similar findings have been reported in the recent literature [58,59,60].

### 3.4. Effect of Solution pH on the Cr(VI) Biosorption

The adsorption of metallic ions from aqueous solutions could be affected by pH-values, (2–11), where the Cr(VI) ions biosorption by the two used biosorbents has been investigated. The maximum removal percentage of Cr(VI)ions by the fungal biomass and the extracellular melanin 98.147% and 98.880%, respectively, was observed at pH 2 (Figure 2D). Obtained results revealed that the increasing pH up to 5 resulted in a Cr(VI) removal percentage of only 79.120% and 77.800% for fungal biomass and the extracellular melanin, respectively. The increase in Cr(VI) removal could be attributed to the pronated surface hydroxyl groups (–OH_2_^+^) from melanin. Subsequently, these protonated hydroxyl groups enable the used biosorbent particles to electrostatically interact with negatively charged Cr_2_O_7_^2−^ [61,62]. Similar findings of decreased Cr(VI) biosorption percentage and biosorption capacity with increasing pH have been reported in other reports for a variety of biosorbents such as *Sinorhizobium* sp. [63], and *Pleurotus ostreatus* [64]. On the other hand, it could be observed that melanin particles have a higher removal percentage and biosorption capacity than native *A*. *pullulans* biomass even at higher pH values. This could be explained by the fact that melanin is a polymer of amino acids that contains indole rings, which are highly electronegative. Additionally, melanin has a strong affinity for positively charged Cr(VI) metal ions [65].

The results presented above clearly demonstrate that extracellular melanin has a higher adsorption capacity than fungus biomass. Sometimes the difference in removal percentage was approximately 20%, and the difference in biosorption capacity was 100 mg/g, due to its unique chemical structure. Melanin is a polymer of amino acids that contains indole rings, which give it its dark color. These indole rings are highly electronegative, which means that they have a strong affinity for positively charged metal ions. Fungus biomass, on the other hand, is composed of a variety of organic compounds, including carbohydrates, proteins, and lipids. These compounds do not have the same strong affinity for positively charged metal ions as indole rings. In addition, melanin is a very porous material, which means that it has a large surface area. This large surface area allows melanin to interact with more metal ions, which results in a higher adsorption capacity.

### 3.5. Biosorption Isotherms

Adsorption isotherms are mathematical equations that demonstrate how the concentration of a metal in a solution at equilibrium relates to how much metal is adsorbed onto a biosorbent. They are used to determine the spreading and interface of metals in the biphasic system at equilibrium. Adsorption isotherms are defined by several parameters, including the initial level of the adsorbate in the aqueous phase, the amount of biosorbent, the relative capabilities of the metal’s adsorption, and competition among the solutes. These parameters are also identified by specific values that reflect the surface properties and ability of a biosorbent for the adsorption of heavy metal ions [65,66].

**Figure 3:**
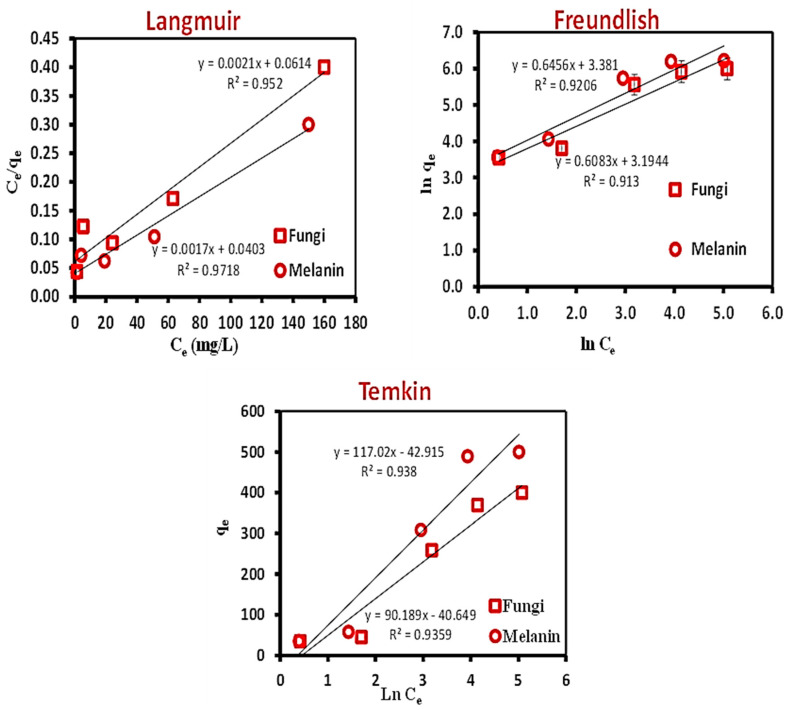
Equilibrium isotherm models; Langmuir, Freundlich, and Temkin for the biosorption of Cr(VI) ions onto the used fungal biomass and the biosynthesized melanin.

In this study, the linear Langmuir, Freundlich, and Temkin models were applied to the experimental data to find the best-fitted model. The linear graphs of the three applied models are illustrated in Figure 3, and their corresponding parameters are tabulated in Table 3.

The relevance of the isotherm equations is evaluated by R^2^. The Langmuir isotherm was further documented by analyzing the Langmuir equilibrium parameter (R_L_), as outlined in Equation (3).

(3) $$R_{L}=\frac{1}{1+K_{{L\;C}_{0}}}\;$$

The calculated R_L_ value was found to be 0.749 and 0.706 for the used fungal biomass and the extracellular melanin, respectively. The biosorption process’s nature is distinguished by the R_L_ value, e.g., unfavorable R_L_ > 1); linear (R_L_ = 1); favorable (0 < R_L_ < 1); irreversible (R_L_ = 0) [67,68].

The obtained R_L_ value indicated that the Cr(VI) biosorption onto the used fungal biomass or the extracellular melanin was a favorable process. The value of 1/n of the Freundlich isotherm model illustrates the probability of the isotherm, e.g., irreversible (1/n = 0); favorable (0 < 1/n < 1); and unfavorable (1/n > 1). The calculated 1/n value (0.608 and 0.646) favored the Freundlich isotherm model. The value of R^2^ was applied to evaluate the fit of both isotherm models. The values were attained to be above 0.90 for both models, identifying that the data of isotherm interpretations fit well for both models. However, the fit was more favorable to the Langmuir model than the Freundlich model [69]. The Temkin isotherm model (Table 3) takes into account the influence of adsorbent-adsorbate interactions on the adsorption process. In accordance with the R^2^ value, the Temkin isotherm model is well-fitted with the investigational data specifying the energetic homogeneity of the biosorption sites and chemisorption process (RT/b lnKT = 0.637 and 0.693 while B = 90.189 and 117.017 mg L^−1^ for the used fungal biomass and the biosynthesized melanin, respectively). The melanin particles have given the best biosorption rate of Cr(VI) metal ion removal.

### 3.6. Biosorption Kinetics

**Figure 4:**
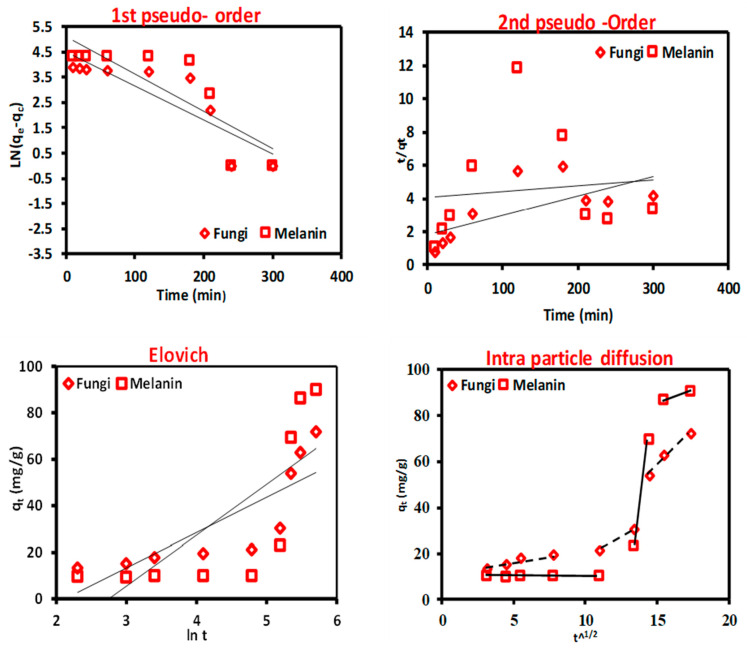
Biosorption kinetics models of Cr(VI) ions onto the used fungal biomass and the biosynthesized melanin particles.

It is important to characterize metal ion biosorption in kinetic terms to understand the behavior involved in biosorption, such as mass transfer and chemical interactions, where, the greatest amount of metal is absorbed in the first minutes to hours owing to the accessibility of free active sites of the biosorbent surface [70,71,72,73]. In this work, four of the most extensively benefitted kinetic models were applied to define the mechanism of Cr(VI) ions onto the treated fungal biomass and the biosynthesized melanin particles pseudo-first-order, pseudo-second-order, Elovich model, and intraparticle diffusion model. The graphical demonstrations of these models and the kinetic data are publicized in Figure 4 and Table 4.

The first-order Lagergren model assumes that the adsorption rate is positively related to the amount of vacant adsorption sites [74]. Previous studies [75,76] have shown that the first-order Lagergren model is not always relevant to all practical data across the biosorption process. Herein, the model is appropriate for depicting the kinetic data of the Cr(VI) ions biosorption, although the correlation coefficient (R^2^ = 0.774 and 0.707 for the used fungal biomass and the biosynthesized melanin, respectively) which is lower than 0.845. Nevertheless, the considered uptake capacity values are respectably matched with the experimental values, also suggesting the suitability of a pseudo-first-order model for describing the kinetics of Cr(VI) ions biosorption onto the used fungal biomass and the biosynthesized melanin, which indicates the physical nature of the adsorption process. These findings are consistent with the previous literature [74].

The pseudo-second-order model posits that the biosorption capacity is directly proportional to the amount of occupied active sites on the biosorbent surface and that the rate-limiting step in biosorption may involve valence forces through sharing or exchanging electrons between the biosorbent and the adsorbate [76]. The low correlation coefficient (R^2^) and the significant difference between the calculated biosorption capacity values (q_calculated_) and the experimental ones (q_experimental_) specify that this model is not appropriate to describe the kinetics of Cr(VI) ions biosorption onto the used biosorbent particles. Elovich kinetic model can be used to identify heterogeneous adsorbents and their role in the adsorption process, but it does not predict any specific adsorption mechanism [42]. The Elovich kinetic model has low R^2^ values, which makes sense because the kinetic data were well-fitted to the pseudo-first-order model. This indicates that the biosorption process is physical, while the Elovich model is more suited to describe chemosorption processes.

The steps that control the biosorption rate were elucidated using the intraparticle diffusion model. The three separate regions with varied slopes (K_id_) and intercepts (C_i_) that did not pass through the origin (Figure 4) revealed that the biosorption process of Cr(VI) ions onto the used biosorbent particles was not influenced by the intra-particle diffusion alone but was also affected by more than one process. The intra-particle diffusion model fits the data well, with a high correlation coefficient (0.900 and 0.844) for the used fungal biomass and the biosynthesized melanin, respectively. Data from Table 5 display a comparison of the biosorption capacity of Cr(VI) ions using fungal biomass and melanin.

### 3.7. Decision Tree Learning Algorithm

The data from the equilibrium isotherms and kinetic studies (Table 6 and Table 7) for both fungal biomass and melanin were used for constructing the DT. For each tree, the data were split into two sets: 70% training and 30% testing. Upon performing the learning process, the predicted and error values mutually with the terminal nodes, were determined for both absorbents (fungal biomass and melanin).

DT begins with a root node and ends up with many branches and roots, i.e., many solutions. The root node is the first node encountered when traversing the tree during the prediction process. From the root node, the decision tree branches into internal nodes (decision nodes) based on different attribute values, and eventually, the leaves (terminal nodes) are reached, providing the final predictions or decisions.

DT creates classifications for the input variables (categorical and/or continuous). As a result, the population can be divided into two or more homogeneous groups. These homogeneous sets are built using the most substantial differentiator on the input variables [27,80]. No information is available about the combination of DT with equilibrium isotherms and kinetic studies for the determination of relative variable importance and modeling the biosorption process. Herein, DT was applied to handle our suggestion regarding identifying the most incredible biosorption parameters and predicting the optimal range of these parameters.

#### 3.7.1. Selection of DT

**Figure 5:**
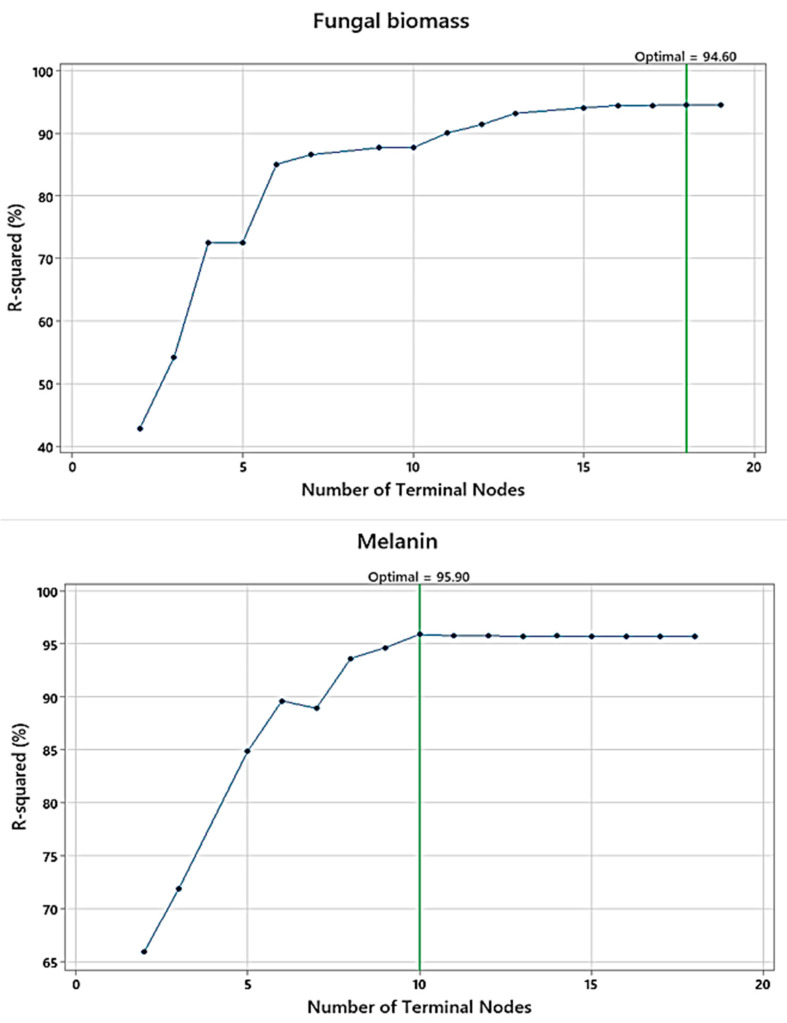
The plots of the number of terminal nodes versus R^2^ generated by a DT for Cr(VI) removal by fungal biomass, and melanin.

The terminal nodes were plotted versus R^2^ (Figure 5) for fungal biomass, and melanin, to identify the smallest regression tree that maximizes the R^2^ value. Regarding Cr(VI) removal by the fungal biomass, 18 terminal nodes represent 18 trees that were produced from the validation samples, whereas for melanin as absorbent, there are 10 terminal nodes and 10 trees. The R^2^ values of the validation samples for both adsorbents exemplary level off as the tree grows. The maximized R^2^ values for the optimal regression tree were 96.26, and 96.70% (for training) and 94.60 and 95.90% (for testing), for fungal biomass and melanin, respectively. These trees had a maximum R^2^ value.

**Figure 6:**
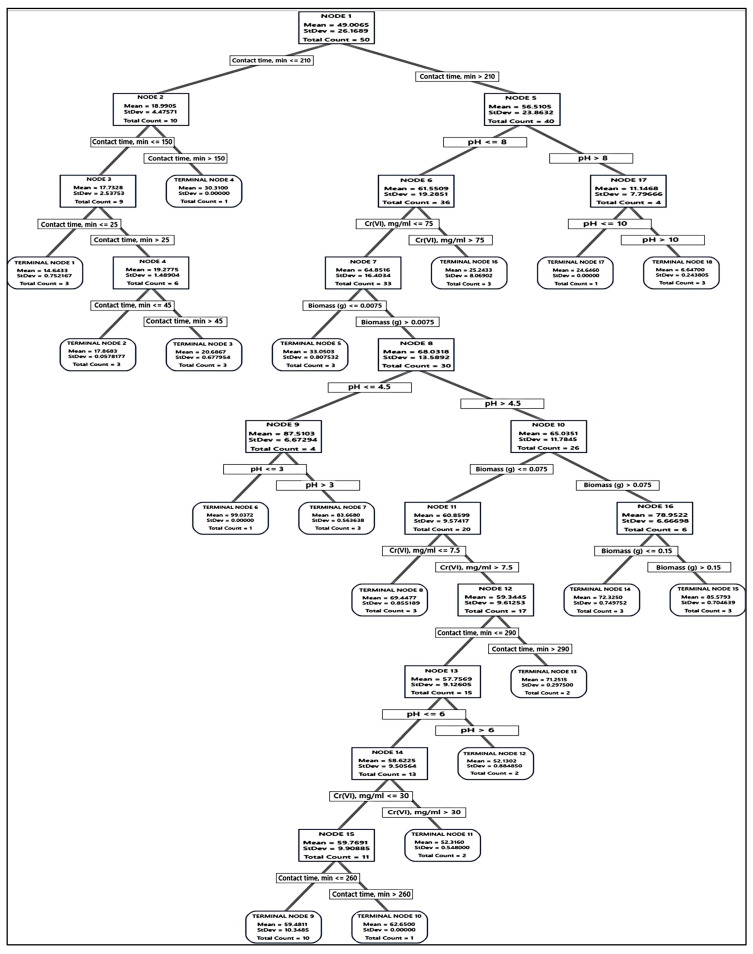
The optimal decision tree diagram of the Cr(VI) removal (%) using fungal biomass. A total of 75 equilibrium isotherms and kinetic data points were employed in the DT to predict the optimum conditions for Cr(VI) removal. The squares contain the data utilized for node selection.

**Figure 7:**
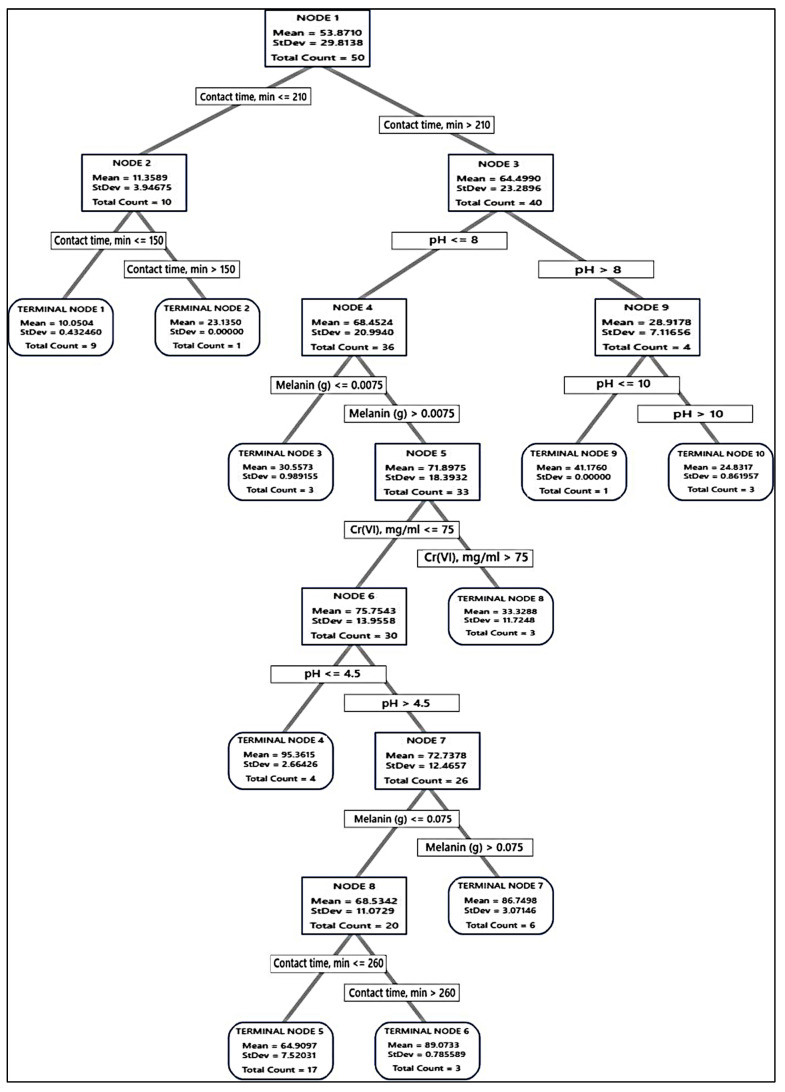
The optimal decision tree diagram of the Cr(VI) removal (%) using melanin. A total of 75 equilibrium isotherms and kinetic data points were employed in the DT to predict the optimum conditions for Cr(VI) removal. The squares contain the data utilized for node selection.

Accordingly, the tree was built and the distinction of terminal nodes on the diagram was explored. The DT diagrams for the two absorbents (Figure 6 and Figure 7) depict all 75 cases from the whole data set. The goal was to discover the greatest Cr(VI) removal means with the fewest standard deviations.

For the fungal biomass, the terminal node No. 6 was the best node, achieving 99.07% Cr(VI) removal, whereas, for melanin, the terminal node No. 4 achieved 95.36% Cr(VI) removal, but this node was not selected because it had large standard deviation, being 2.66, instead, the terminal node No. 6 (89.07% Cr(VI) removal) was selected as the best terminal node of the DT. These nodes had a maximum R^2^ value with a standard deviation of less than 1. 

Regarding the absorption of Cr(VI) by fungal biomass, the first node started with 50 cases and split at two branches of contact time, i.e., ≤210 and >210 min. The latter was the best, and split to pH > 8 and ≤ 8, the latter was also split based on initial Cr(VI) into the terminal node of Cr(VI) > 75 mg/mL, and node 6 Cr(VI) ≤ 75 mg/mL, which subdivided into terminal node 4 of fungal biomass ≤ 0.0075 g, and node 7 of fungal biomass > 0.0075 g, leading finally to optimum terminal node 5, the node rules were contacting time > 210 min, initial Cr(VI) ≤ 75 mg/mL, fungal biomass > 0.0075 g, and pH ≤ 3 with the highest Cr(VI) removal (99.04%, with lowest SD value = 0.5393), followed by the terminal node 15 (85.58%, with SD value = 0.7046). The other terminal nodes did not show further improvements. Alternatively, terminal node 1 has the smallest Cr(VI) removal (14.64%), suggesting that the data in the terminal node are probably skewed.

Regarding the absorption of Cr(VI) by melanin, such as fungal biomass DT, the first node uses 50 cases and is split at two levels of contact time, i.e., ≤210 and >210 min. The latter was split into pH > 8 and ≤8, and the latter was split into several nodes and terminal nodes, where terminal node 4 was the best in Cr(VI) removal (95.36%) of the entire DT. However, this point was not a suitable one since it recorded a high value of SD (2.6643). Node 6 has been the second, recording (89.04%), with a SD value of 0.7856, which is less than the overall SD. The role of the terminal node was contact time, min > 260, Cr(VI) ≤ 75 mg/mL, 0.0075 < melanin (g) ≤ 0.075, 4.5 < pH <= 8. No further improvements could be observed on the DT terminal nodes.

#### 3.7.2. Evaluation of the DT Models

The error statistics of both DT models were calculated (Table 8). The accuracy of the resulting two trees was specified with high R-squared, and low values of the error measurements (RMSE, MSE, MAD, and MAPE) for training and testing processes. However, it is obvious that the four tested parameters in the two DTs were important predictors, furthermore, the DT of fungal biomass has a lower error rate in comparison to the DT of melanin, this conclusion is conversely to that obtained by the equilibrium isotherms and kinetics data, suggesting that DT can help improve the accuracy of Cr(VI) removal by discovering the optimum overall situation sittings that maximize the biosorption process.

#### 3.7.3. Relative Importance of the Variable

**Figure 8:**
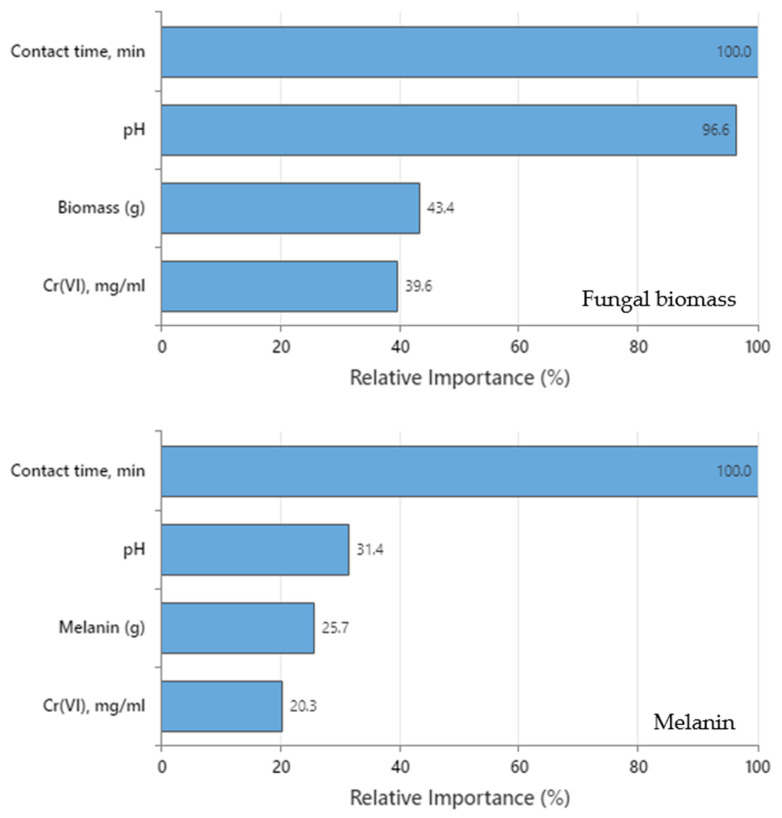
The relative importance of the 4 tested parameters on Cr(VI) removal by fungal biomass or melanin as inferred by the DT.

The relative importance of the variables for both DTs was computed as the increasing percentage relative to the leading variable. Figure 8 shows that the four predictors in both DTs are important to the tree. Contact time was the leading predictor in both trees (fungal biomass and melanin) since it has a relative importance of 100% contribution in Cr(VI) removal. The other variables are standardized concerning the most important predictor. As a result, the significance of each variable can be simply interpreted. The leading variable is often identified as the one with the maximum improvement score (and hence regarded as 100% critical), and the remaining variables are sorted consequently [9]. Surrogate or primary splitters in the DT are considered crucial variables. The most essential variable is always given 100% relative importance, while the non-important variable is not represented in the tree. Regarding fungal biomass DT, relative importance in descending order was contact time (100%) > pH (96.6%) > fungal biomass dose (43.4) > initial Cr(VI) concentration (39.6%), whereas for melanin DT was contact time (100%) > pH (31.4%) > melanin dose (25.7%) > initial Cr(VI) concentration (20.3%).

Despite the positive importance of the four variables for both DTs, the relative importance discloses that contact time must be strongly considered to be accurately regulated and kept tracked during the Cr(VI) removal process. The role of contact time was more obvious in melanin DT than in fungal biomass DT. Determination of the relative importance values can assist in deciding which variables should be regulated, monitored, or excluded. In this assembly, the initial Cr(VI) concentration in melanin DT has the lowest relatively important value (20.3%) concerning contact time. This conclusion comes in line with the goal of the present work, in which the current DTs show efficient accuracy in the detection of these kinds of hidden relationships among tested parameters compared to the equilibrium isotherms and kinetic studies.

#### 3.7.4. Validation of DT Models

Both DT models were experimentally validated. The forecast levels of each of the four tested parameters were determined at various nodes (Table 9). The laboratory validation experiments were carried out in triplicate to validate the procedure conditions estimated from both DT models. When compared to projected values, the experimental Cr(VI) removal date corroborated and validated the fitness of both models. At various nodes, the Cr(VI) removal by both models was confirmed. The speculative values of the ideal levels that maximize Cr(VI) removal by the four variables were also validated. The only exception was nodes number 4 (86.04%), and 7 (80.21%) in the DT model of melanin, which showed shifting by −9, and −6.5% in comparison to the predicted levels (95.36 and 86.75%), respectively. This is expected and can be attributed to the high value of the standard deviation of the node (Figure 7). Interestingly, DT modeling of fungal biomass was found to be better than melanin in the Cr(VI) removal process. One other positive outcome of the current validation is that both DTs effectively introduce more alternative solutions for the Cr(VI) removal process, in comparison to the equilibrium isotherms and kinetic data.

### 3.8. Surface Topology, and Chemistry of the Biosorbents

#### 3.8.1. FT-IR Spectral Analysis

**Figure 9:**
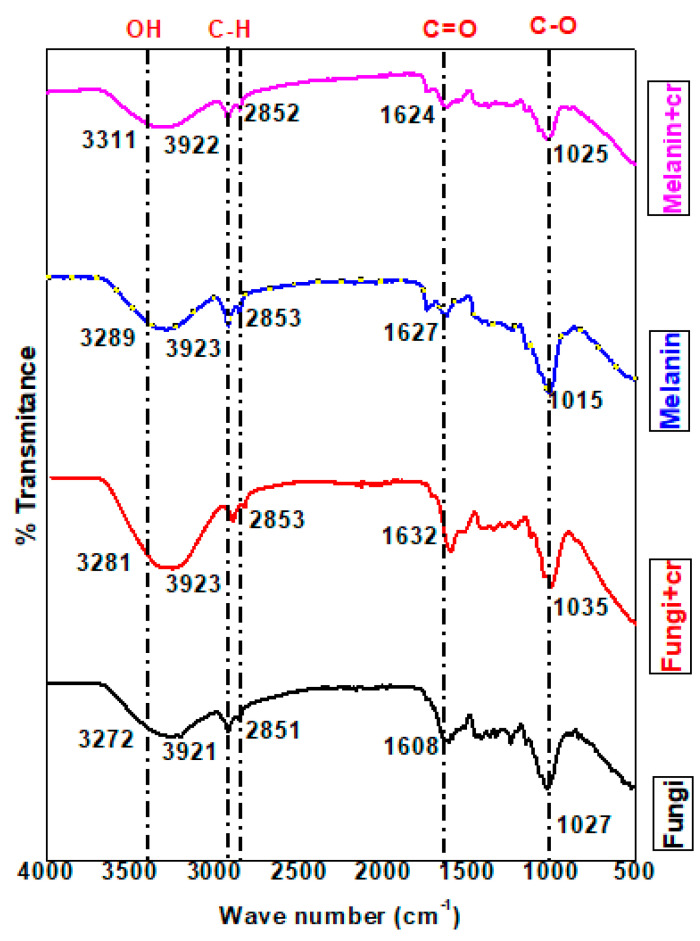
The FT-IR spectral chart of the used fungal biomass and its extracellular melanin before and after Cr(VI) biosorption.

The FT-IR analysis was applied to explore the vibrations of the characteristic functional groups of the fungi biomass, and melanin as well as chromium ions adsorbed on the surface of fungi biomass, and melanin [79]. The results of FT-IR spectral analysis (Figure 9 and Table S1) for the pure fungi powder (F) revealed the appearance of an absorption band ascribed to a medium N-H or OH stretching group identified at ν = 3272.99 cm^−1^ with a shift recorded in the value of the same group for chromium adsorbed on fungi biomass (F-Cr) at ν = 3281.85 cm^−1^. The stretching absorption bands of OH or N-H stretching group in the case of melanin (M) and chromium adsorbed on melanin (M-Cr) were specified at wavenumbers (ν) 3289.57, and 3311.59 cm^−1^, respectively. A previous study supported that this range of wavenumbers is related to OH groups [79].

The low to high shift in values of peak wavenumbers is ascribed to the binding of chromium to the -OH and –NH functional groups [81]. The wavenumber value in the case of M-Cr was shifted from the value of the same group in the analysis of melanin (M). The strong broad absorption band due to C-H stretching appeared in the analysis of all samples with a slight shift in the case of chromium ions adsorbed on fungi, and melanin surfaces within ν = 2921.32–2923.82 cm^−1^. The shifted intensity of the C-H stretching peak specified the binding of Cr(VI) to the C-H group of melanin, and fungi biomass. The FT-IR data verified the distinctive absorption bands attributed to the sp3 C-H stretching group at ν = 2851.54 cm^−1^ (F), attended by a slight shift for F-Cr at ν = 2853.71 cm^−1^, ν = 2853.50 cm^−1^ (M), and ν = 2852.89 cm^−1^ (M-Cr). Specifically, a strong stretching absorption band was noticed in the FT-IR analysis of melanin at ν = 2323.61 cm^−1^ is ascribed to the O=C=O group, and disappeared in the analysis of M-Cr. This result supported the participation of the O=C=O group in the adsorption process of chromium ions on the melanin surface.

Similarly, absorption bands ascribed to the stretching C-H group appeared in the analysis of melanin within ν = 2170.03–2011.0 cm^−1^ along with the disappearance of these absorption bands in the analysis of M-Cr. The data of the melanin sample referred to the presence of a stretching carbonyl ester group at ν = 1740.66 cm^−1^ with a slight shift in the value of this band at ν = 1740.82 cm^−1^ in the analysis of M-Cr. The fact that the shifted values of carbonyl groups reinforced the contribution of this group in the absorption of chromium ions. Alternatively, a strong absorption band related to stretching amide carbonyl group was noticed in all samples at ν = 1608.82 cm^−1^ (F), ν = 1632.11 cm^−1^ (F-Cr), ν = 1627.26 cm^−1^ (M), and ν = 1624.12 cm^−1^ (M-Cr). Predominantly, the FT-IR data of fungi sample indicated the presence of absorption bands at ν = 1589.78, and 1513.92 cm^−1^ owing to medium bending N-H groups. The disappearance of absorption bands in these regions in the analysis of F-Cr verified the adsorption of chromium ions on the fungi surface with the aid of N-H groups. The FT-IR analyses of melanin, and Cr-adsorbed on melanin surface revealed the absence of the characteristic absorption bands in the regions of N-H groups [78]. In addition, bending O-H groups appeared in the analysis of fungi within ν = 1416.81–1330.35 cm^−1^, while these values were intensively shifted in the analysis of F-Cr (ν = 1443.78–1315.49 cm^−1^). The analysis of melanin revealed medium absorption bands at ν = 1413.49, and 1370.51 cm^−1^ attributed to the bending O-H group, although one of these bands disappeared, and the other absorption band was shifted at ν = 1374.93 cm^−1^ in the analysis of M-Cr. The stretching C-N groups were documented for all samples with a slightly shifted value at ν = 1244.81 (F), ν = 1248.68 (F-Cr), ν = 1232.96 (M), and ν = 1238.20 cm^−1^ (M-Cr). The shift of peak to higher wavenumber values indicated the chemisorption of Cr(VI) to the functional groups. Our results are in accordance with the previous report concerning the interpretation of the C-N group, and the increased shift in intensity [79]. Correspondingly, stretching absorption bands assigned for Cr=O groups appeared at ν = 1035.11 cm^−1^ (F-Cr), and for M-Cr at ν = 1025.67 cm^−1^ indicating the adsorption of chromium as dichromate anions on the surface of fungi biomass and melanin [82,83]. An absorption band at ν= 840.55 cm^−1^ was established for strong bending vibration of the C-H group in the FT-IR analysis of melanin, this absorption band disappeared in the analysis of F-Cr. The disappearance of the C-H group is related to the involvement of this group in the adsorption of chromium ions on the fungi biomass. The spectral of FT-IR revealed absorption bands at ν = 525.47, 471.52 (F), ν = 486.78 (F-Cr), ν = 482.99, and 425.76 (M) owing to phenyl rings, while this band was not recorded for the sample of M-Cr. Cr(VI) ions predominantly bind to fungi biomass, and melanin as negatively charged HCrO_4_^−1^ and Cr_2_O_4_^−2^ groups to positively charged sites or functional groups of fungi, and melanin. The introduction of oxygen and hydrogen atoms in the adsorbed complex of Cr(VI) might be the cause for increased perceived % transmittance [84]. The interpretations of the FT-IR verified that the adsorbent has active sites for Cr(VI) adsorption.

#### 3.8.2. SEM Investigation

**Figure 10:**
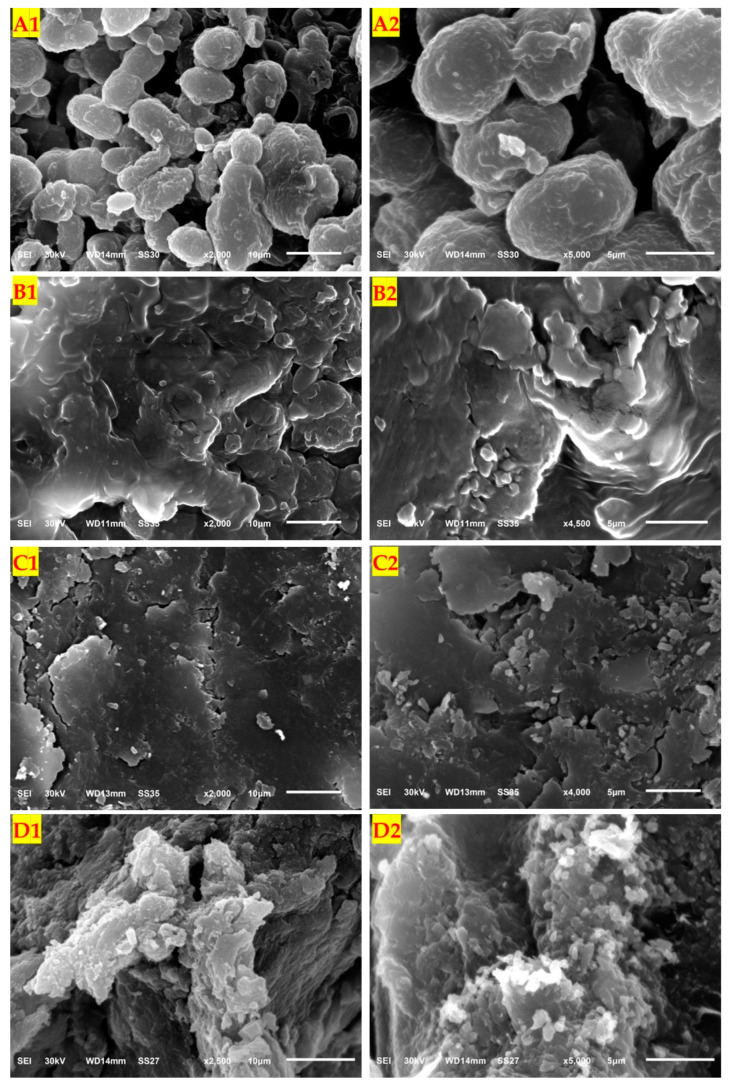
SEM photographs of the fungi biomass and melanin before and after Cr(VI) biosorption, showing fungal biomass before (**A1**,**A2**) and after (**B1**,**B2**) adsorption, as well as melanin particles before (**C1**,**C2**), after (**D1**,**D2**) adsorption.

The SEM image of biomass cells of *A. pullulans* AKW, and its melanin particles were investigated against Cr(VI) ions using two different magnification powers. The Cr(VI)-unloaded biomass cells of *A. pullulans* Akw, showed to be characterized by the regular and homogenous shape of the cells (Figure 10(A1,A2)). In the treated fungal biomass with Cr(VI), detectable alterations in morphology (cell shape, size, and surface features), and also indentation in cells, clear deformation, shrinking of cells, flighting, and some distortion, with enlargement, could be noticed (Figure 10(B1,B2)). Our results are in harmony with the previous findings that pointed out that the uptake and accumulation of heavy metals by fungal cells, caused morphological alteration, physiological damage, molecular disturbance, ultrastructural changes, and inhibition of the antioxidant system [85,86]. Similarly, the SEM images of extracellular melanin particles before and after the adsorption process of Cr(VI) were investigated. The controlled sample of melanin particles (Figure 10(C1,C2)) appeared to be more amorphic and opaque, while, the treated melanin (Figure 10(D1,D2)) showed flourished amorphous particles as a result of Cr(VI) ions. The image showed also changes in the aggregation pattern and the surface appearance of the melanin particles, including texture, shape, and aggregation of the particles. Similar conclusions were reported during the examination of the structure of natural and synthetic eumelanin using SEM [87].

Melanin and fungal biomass have better morphology to adsorb Cr(VI), and their surfaces are rich with porous which acquire Cr(VI). This provides favorable conditions for the bioadsorption process of organic pollutants [88]. The adsorbent surface of melanin and fungal biomass profoundly adsorbed Cr(VI) ions in the inner walls.

#### 3.8.3. EDX Analyses

The analysis of fungal biomass revealed the presence of carbon (75.97%), and oxygen (23.32%) as the main elements, along with rare elements such as chlorine, potassium, calcium, copper, and zinc. The analysis of Cr(VI) adsorbed on fungal biomass indicated the adsorption of Cr(VI) with 1.39% of weight %, and 0.35% of atomic percentage. Hereafter, it is established that the treatment of melanin, and fungal biomass with Cr(VI) solution does not destroy any of the basic functional groups of the adsorbent [89].

**Figure 11:**
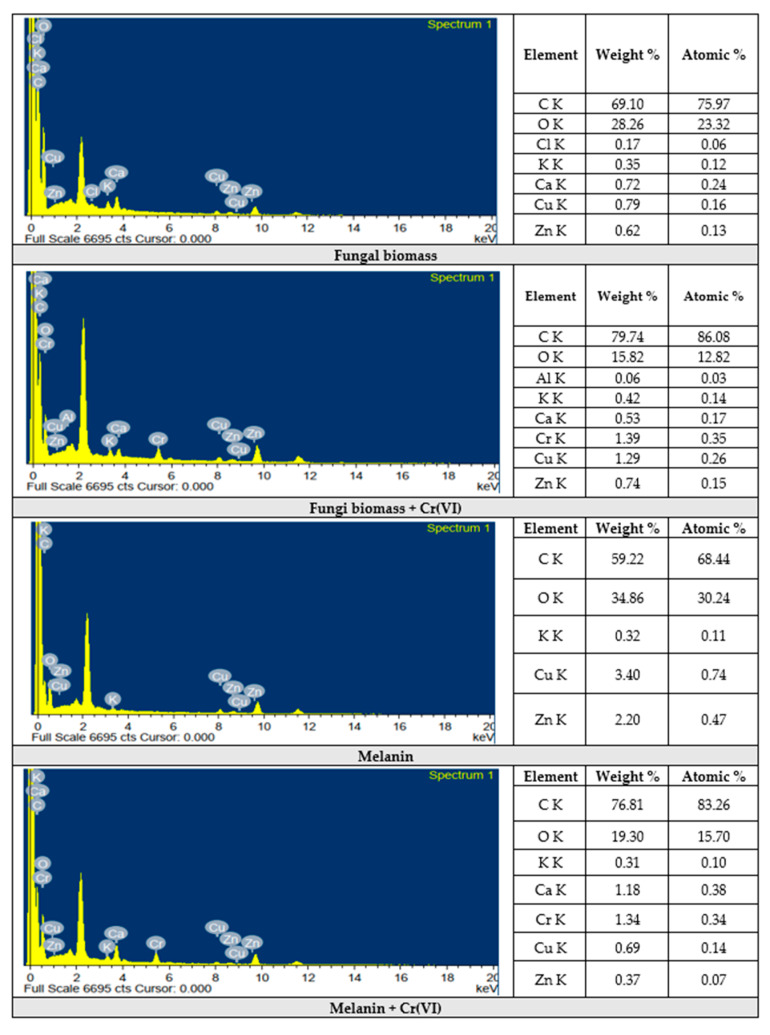
EDX-analyses of the fungi biomass and melanin before and after Cr(VI) biosorption.

The images of the EDX analyses of melanin, fungal biomass, and Cr(VI) ions adsorbed on their surfaces are shown in Figure 11. The results of melanin indicated the presence of carbon (68.44%), and oxygen (30.24%) as the major elements with rare atomic percentages for potassium, copper, and zinc. The melanin-adsorbed Cr(VI) displayed the presence of Cr(VI) with 1.34% of the weight and 0.34% of atomic percentages. Similarly, the analyses were applied for fungi biomass, and melanin as well as chromium ions adsorbed on their surface to examine the possible adsorption of Cr(VI) ions on their surfaces [79]. Our results are in line with the results obtained by [90].

Based on the current studies, it is supposed that Cr(VI) may interact with the surface of the biomass or melanin through a variety of mechanisms, including hydrophobic interactions, electrostatic interactions, and hydrogen bonding.

### 3.9. Interaction Mechanism of Cr(VI) Ions onto the Biosorbent Particles

**Figure 12:**
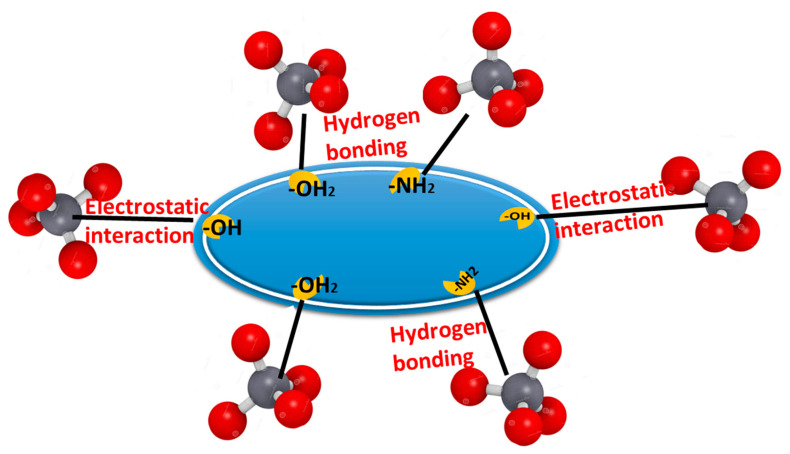
Binding mechanism of Cr(VI) ions onto the used biosorbent particles.

The biosorption of Cr(VI) is affected by the nature of the functional groups in the metal ion, the textural and surface properties of the used biosorbent materials, the diffusion of the metal ion molecules to the used biosorbent, and how the metal ion molecules interact with the used material particles. The surface of the used biosorbent particles has pronated hydroxyl groups (–OH_2_^+^) and (-NH_2_), which enable them to electrostatically interact with negatively charged Cr_2_O_7_^2−^. Moreover, the abundant hydroxyl groups are highly capable of forming hydrogen bonds with CrO_4_^2−^, and HCrO_4_^−^. Taken collectively, the combination of hydrogen bonding and electrostatic attraction could maximize the biosorption of Cr(VI) onto the used biosorbent particles. The removal percentage of Cr(VI) ions increased under acidic conditions, while decreasing under basic conditions, indicating that electrostatic attraction plays a critical role in the biosorption of negative Cr(VI) ions onto the used biosorbent particles surface (Figure 12).

## 4. Conclusions

The comparative study involving fungus, *A*. *pullulans* AKW biomass and its extracellular melanin particles as biosorbent agents for Cr(VI) ions was conducted. The parameters of contact time, initial concentration of Cr(VI), biosorbent dosages (fungal biomass or melanin), and pH with their influence on the biosorption process of Cr(VI) ions were investigated. Equilibrium isotherms were applied for experimental data. Langmuir model was shown to be more favorite for the biosorption process compared to the Freundlich isotherm. As well as, the Temkin isotherm fit well with the experimental data, indicating the energetic homogeneity of the biosorption sites and chemisorption process for fungus biomass and melanin. Regarding the kinetic studies, the biosorption process obeyed pseudo-first-order rather than pseudo-second-order. The DTs of both fungal biomass and melanin were constructed. The DT of fungal biomass was a lower error compared to melanin. Dts have efficiency in optimizing Cr(VI)removal compared to equilibrium isotherms and kinetic study. The FT-IR spectra of melanin, and fungal biomass verified the adsorption of Cr(VI) on their surface as appeared from the obtained data with a notable shift in the values of the stretching vibrations of most of the functional groups. The C-H stretching, stretching of the N-H bond, and “O-H” hydroxyl groups produced the shifting of absorption bands in the FT-IR spectra attributed to the biosorption of Cr(VI). The SEM and EDX analyses supported the reality that melanin and fungal biomass can remove heavy metals leading to detoxification. Summing up, the extracellular melanin was found to be more efficient in the biosorption process of Cr(VI) ions compared to fungus biomass, it could be applicable on a large scale since its feasibility of production by *A. pullulans* AKW, using a cheap medium.

Based on the data of the current work, further research can focus on applying the DT algorithm modeling Cr(VI) ions removal using a combination of fungal biomass and melanin in biosorption strategies. Synergistic effects between different biosorbents may enhance the overall efficiency and versatility of the process. Likewise, combining another approach of machine learning such as artificial neural networks could lead to enhanced efficiency and uptake capacity of the biosorbents, making them more attractive for practical applications. Furthermore, the generalization of the modeling process in the current study to include other sorbent/absorbate interactions. Moreover, the scenario of the performance of fungal biomass and melanin under real-world conditions will provide valuable information for their implementation. However, it is recommended to assess the toxicity and potential environmental impact of using fungal biomass and/or melanin for Cr(VI) removal during the biosorption process.

## Figures and Tables

**Table 1:** Equations of Langmuir, Freundlich, and Temkin models for biosorption of Cr(VI) onto the used fungal biomass and the biosynthesized melanin [39].

Adsorption Models	Equation	Parameter
Langmuir	$\frac{C_{e}}{q_{e}\;}=\frac{1}{q_{m}K}+\frac{C_{e}}{q_{m}}\;\;\;\;\;\;\;\;\;$	q_e_ is the amount of Cr(VI) ions biosorbent at equilibrium (mg/g), q_m_ is the supreme monolayer coverage aptitudes (mg/g), K is the Langmuir constant (L/mg), and C_e_ is the equilibrium concentration of Cr(VI) ions (mg/L).
Freundlich	$ln\;q_{e}=ln\;K_{f}+\frac{1}{n_{f}}\;\;ln\;C_{e}\;\;\;$	q_e_ is the Cr (VI) ions amount biosorbent at equilibrium (mg/g); C_e_ is the Cr (VI) ions equilibrium concentration (mg/L); and K_F_ and n_f_ are Freundlich constants related to the biosorption aptitude and biosorption intensity, respectively
Temkin	*q_e_ = B ln KT + B ln C_e_*	K_T_ is the Temkin constant referring to equilibrium maximum binding energy and B is the Temkin constant interrelated to bio-sorption heat.

**Table 3:** The biosorption of Cr(VI) onto the used fungal biomass or the biosynthesized melanin at 25 °C based on Langmuir, Freundlich, and Temkin parameters.

Isotherm Parameters	Fungal Biomass	Melanin
Langmuir		
q_m_ (mg g^−1^) calculated	485.747	595.974
K_L_ (mg L^−1^)	0.034	0.042
R^2^	0.976	0.986
Freundlich		
K_F_ (mgL^−1^/n L1/n g^−1^)	24.395	1.907
Nf	1.64	1.55
R^2^	0.956	0.959
Temkin B (mg L^−1^)	90.189	117.017
K_T_ (KJ mol^−1^)	0.637	0.693
R^2^	0.967	0.969

**Table 4:** Kinetic models for biosorption of Cr(VI) ions onto the used fungal biomass and the biosynthesized melanin particles.

Kinetic Model	Fungal Biomass		Melanin
Pseudo-first-order	q_e_ (mg/g) Calculated	69.4148	163.531
	q_e_ (mg/g) Experimental	62.9	86.400
	k_1_ (min^−1^)	−0.015	0.015
	R^2^	0.774	0.707
Pseudo-second-order	q_e_ (mg/g) Calculated	85.825	107.704
	q_e_ (mg/g) Experimental	62.9	86.400
	k_2_ (g/mg min)	0.01	0.0048
	R^2^	0.678	0.235
Elovich	ß (g/mg)	15.137	21.853
	ὰ (mg/g min)	−32.062	−60.041
	R^2^	0.820	0.692
Intra-particle diffusion	K_1,_	3.888	5.748
	C_1_	−5.933	−23.697
	R^2^	0.900	0.844

**Table 5:** Comparison of the maximum biosorption capacity of Cr(VI) ions onto the used fungal biomass and the extracellular melanin particles with those of previous studies.

Adsorbent	qm (mg/g)	Reference
Fungal biomass and melanin	485.747 and 595.974	Current study
Removal of Cr(VI) by polyethyleneimine-impregnated activated carbon	114	[77]
Biosorption of chromium metal ions onto *Ludwigia stolonifera*	43.478	[39]
Biosorption of Cr(VI) by *Bacillus megaterium* and *Rhodotorula* sp. inactivated biomass	34.80	[56]
Melanin-embedded materials effectively remove Cr(VI)	19.60 and 6.24 for IMB and CMB	[78]
Melanin nano pigment from *Pseudomonas stutzeri*	126.9	[79]
Equilibrium and kinetic studies of copper(II) removal by fungal biomasses	7.74 and 12.08	[74]

**Table 6:** The array equilibrium isotherms and kinetic data for maximization of Cr(VI) removal by the biomass of *A. pullulans*, and the predicted and error values as well as terminal nodes of the DT at each data point.

Isotherm or Kinetic Test	Run	Tested Parameter				Cr(VI) Removal, %					
						Actual		Decision Tree			
		Time, min	Cr(VI), mg/mL	Biomass (g/100 mL)	pH	Value	Mean	Type	Fitted	Error	Terminal Node
Contact time	1	10	10	0.010	5	13.44	13.46 ± 0.11	Test	14.64	−1.20	1
	2	20	10	0.010	5	15.41	15.25 ± 0.14	Test	14.64	0.77	1
	3	30	10	0.010	5	17.88	17.87 ± 0.07	Training	17.87	0.01	2
	4	60	10	0.010	5	19.35	19.56 ± 0.19	Test	20.69	−1.34	3
	5	120	10	0.010	5	21.11	21.24 ± 0.15	Training	20.69	0.42	3
	6	180	10	0.010	5	29.23	30.35 ± 1.15	Test	30.31	−1.08	4
	7	240	10	0.010	5	53.00	54.02 ± 1.01	Training	59.48	−6.48	9
	8	280	10	0.010	5	62.50	62.99 ± 0.72	Test	62.65	−0.15	10
	9	300	10	0.010	5	70.95	71.82 ± 1.03	Training	71.25	−0.30	13
Initial Cr(VI)	10	240	5	0.010	5	68.25	69.45 ± 1.05	Training	69.45	−1.19	8
	11	240	10	0.010	5	53.34	54.08 ± 0.79	Training	59.48	−6.15	9
	12	240	50	0.010	5	50.45	51.70 ± 1.21	Test	52.32	−1.86	11
	13	240	100	0.010	5	35.96	36.87 ± 1.03	Test	25.24	10.72	16
	14	240	200	0.010	5	19.43	20.03 ± 0.86	Training	25.24	−5.81	16
Melanin	15	240	10	0.005	5	33.04	33.05 ± 0.99	Training	33.05	−0.01	5
	16	240	10	0.010	5	50.84	50.61 ± 0.65	Training	59.48	−8.64	9
	17	240	10	0.050	5	59.54	60.26 ± 0.81	Training	59.48	0.06	9
	18	240	10	0.100	5	71.59	72.33 ± 0.92	Training	72.33	−0.74	14
	19	240	10	0.200	5	85.32	85.58 ± 0.86	Training	85.58	−0.26	15
pH	20	240	10	0.010	2	98.25	98.16 ± 0.94	Test	99.04	−0.78	6
	21	240	10	0.010	4	82.88	83.67 ± 0.69	Training	83.67	−0.79	7
	22	240	10	0.010	5	79.32	79.16 ± 1.06	Test	59.48	19.84	9
	23	240	10	0.010	7	52.32	52.19 ± 0.89	Test	52.13	0.19	12
	24	240	10	0.010	9	23.87	24.61 ± 0.73	Test	24.65	−0.78	17
	25	240	10	0.010	11	6.53	6.65 ± 0.30	Training	6.65	−0.11	18
Contact time	1	10	10	0.010	5	13.36		Test	14.64	−1.28	1
	2	20	10	0.010	5	15.15		Training	14.64	0.51	1
	3	30	10	0.010	5	17.94		Training	17.87	0.07	2
	4	60	10	0.010	5	19.73		Training	20.69	−0.96	3
	5	120	10	0.010	5	21.22		Training	20.69	0.53	3
	6	180	10	0.010	5	31.52		Test	30.31	1.21	4
	7	240	10	0.010	5	54.04		Test	59.48	−5.44	9
	8	280	10	0.010	5	62.65		Training	62.65	0.00	10
	9	300	10	0.010	5	72.97		Test	71.25	1.71	13
Initial Cr(VI)	10	240	5	0.010	5	69.88		Training	69.45	0.43	8
	11	240	10	0.010	5	54.92		Training	59.48	−4.56	9
	12	240	50	0.010	5	51.77		Training	52.32	−0.55	11
	13	240	100	0.010	5	37.99		Test	25.24	12.74	16
	14	240	200	0.010	5	19.64		Training	25.24	−5.60	16
Melanin	15	240	10	0.005	5	32.07		Training	33.05	−0.99	5
	16	240	10	0.010	5	49.88		Training	59.48	−9.61	9
	17	240	10	0.050	5	60.09		Test	59.48	0.61	9
	18	240	10	0.100	5	72.03		Training	72.33	−0.29	14
	19	240	10	0.200	5	84.88		Training	85.58	−0.70	15
pH	20	240	10	0.010	2	97.17		Test	99.04	−1.86	6
	21	240	10	0.010	4	83.99		Training	83.67	0.32	7
	22	240	10	0.010	5	78.03		Training	59.48	18.55	9
	23	240	10	0.010	7	51.25		Training	52.13	−0.88	12
	24	240	10	0.010	9	24.65		Training	24.65	0.00	17
	25	240	10	0.010	11	6.42		Training	6.65	−0.22	18
Contact time	1	10	10	0.010	5	13.58		Training	14.64	−1.06	1
	2	20	10	0.010	5	15.20		Training	14.64	0.56	1
	3	30	10	0.010	5	17.79		Training	17.87	−0.07	2
	4	60	10	0.010	5	19.60		Test	20.69	−1.09	3
	5	120	10	0.010	5	21.40		Test	20.69	0.71	3
	6	180	10	0.010	5	30.31		Training	30.31	0.00	4
	7	240	10	0.010	5	55.02		Test	59.48	−4.46	9
	8	280	10	0.010	5	63.81		Test	62.65	1.16	10
	9	300	10	0.010	5	71.55		Training	71.25	0.30	13
Initial Cr(VI)	10	240	5	0.010	5	70.21		Training	69.45	0.77	8
	11	240	10	0.010	5	54.00		Training	59.48	−5.48	9
	12	240	50	0.010	5	52.86		Training	52.32	0.55	11
	13	240	100	0.010	5	36.65		Training	25.24	11.41	16
	14	240	200	0.010	5	21.02		Test	25.24	−4.22	16
Melanin	15	240	10	0.005	5	34.04		Training	33.05	0.99	5
	16	240	10	0.010	5	51.12		Test	59.48	−8.36	9
	17	240	10	0.050	5	61.13		Training	59.48	1.65	9
	18	240	10	0.100	5	73.35		Training	72.33	1.03	14
	19	240	10	0.200	5	86.54		Training	85.58	0.96	15
pH	20	240	10	0.010	2	99.04		Training	99.04	0.00	6
	21	240	10	0.010	4	84.14		Training	83.67	0.47	7
	22	240	10	0.010	5	80.13		Training	59.48	20.65	9
	23	240	10	0.010	7	53.02		Training	52.13	0.88	12
	24	240	10	0.010	9	25.32		Test	24.65	0.67	17
	25	240	10	0.010	11	6.99		Training	6.65	0.34	18

**Table 7:** The array equilibrium isotherms and kinetic data for maximization of Cr(VI) removal by the melanin of *A. pullulans*, and the predicted and error values, as well as terminal nodes of the DT at each data point.

Isotherm or Kinetic Test	Run	Tested Parameters				Cr(VI) Removal, %					
						Actual		Decision Tree			
	No.	Time, min	Cr(VI), mg/mL	Melanin (g/100 mL)	pH	Value	Mean	Type	Fitted	Error	Terminal Node
Contact time	1	10	10	0.010	5	9.95	9.84 ± 0.14	Test	10.05	−0.10	1
	2	20	10	0.010	5	9.32	9.46 ± 0.24	Test	10.05	−0.73	1
	3	30	10	0.010	5	10.45	10.09 ± 0.45	Training	10.05	0.40	1
	4	60	10	0.010	5	10.36	10.13 ± 0.49	Test	10.05	0.31	1
	5	120	10	0.010	5	10.73	10.11 ± 0.59	Training	10.05	0.68	1
	6	180	10	0.010	5	22.21	23.14 ± 0.93	Test	23.14	−0.92	2
	7	240	10	0.010	5	68.23	69.40 ± 1.16	Training	64.91	3.32	5
	8	280	10	0.010	5	84.55	86.41 ± 1.79	Test	89.07	−4.52	6
	9	300	10	0.010	5	89.05	90.04 ± 0.99	Training	89.07	−0.02	6
Initial Cr(VI)	10	240	5	0.010	5	70.12	70.39 ± 0.95	Training	64.91	5.21	5
	11	240	10	0.010	5	57.18	58.14 ± 0.97	Training	64.91	−7.73	5
	12	240	50	0.010	5	61.49	61.72 ± 0.88	Test	64.91	−3.42	5
	13	240	100	0.010	5	48.88	48.91 ± 0.94	Test	33.33	15.55	8
	14	240	200	0.010	5	26.00	25.05 ± 0.95	Training	33.33	−7.33	8
Melanin	15	240	10	0.005	5	30.20	30.56 ± 1.21	Training	30.56	−0.36	3
	16	240	10	0.010	5	52.22	52.28 ± 0.91	Training	64.91	−12.69	5
	17	240	10	0.050	5	66.95	66.96 ± 1.00	Training	64.91	2.04	5
	18	240	10	0.100	5	83.90	83.77 ± 0.95	Training	86.75	−2.85	7
	19	240	10	0.200	5	89.65	89.73 ± 0.89	Training	86.75	2.90	7
pH	20	240	10	0.010	2	98.76	98.83 ± 0.98	Test	95.36	3.40	4
	21	240	10	0.010	4	93.88	93.87 ± 0.90	Training	95.36	−1.49	4
	22	240	10	0.010	5	77.69	77.82 ± 0.92	Test	64.91	12.78	5
	23	240	10	0.010	7	65.12	65.59 ± 1.02	Test	64.91	0.21	5
	24	240	10	0.010	9	40.25	40.21 ± 0.98	Test	41.18	−0.93	9
	25	240	10	0.010	11	24.85	24.83 ± 1.06	Training	24.83	0.02	10
Contact time	1	10	10	0.010	5	9.68		Test	10.05	−0.37	1
	2	20	10	0.010	5	9.73		Training	10.05	−0.32	1
	3	30	10	0.010	5	10.23		Training	10.05	0.18	1
	4	60	10	0.010	5	10.45		Training	10.05	0.40	1
	5	120	10	0.010	5	10.06		Training	10.05	0.01	1
	6	180	10	0.010	5	24.07		Test	23.14	0.94	2
	7	240	10	0.010	5	69.42		Test	64.91	4.51	5
	8	280	10	0.010	5	88.12		Training	89.07	−0.95	6
	9	300	10	0.010	5	91.03		Test	89.07	1.96	6
Initial Cr(VI)	10	240	5	0.010	5	71.46		Training	64.91	6.55	5
	11	240	10	0.010	5	58.13		Training	64.91	−6.78	5
	12	240	50	0.010	5	60.98		Training	64.91	−3.93	5
	13	240	100	0.010	5	47.99		Test	33.33	14.66	8
	14	240	200	0.010	5	24.11		Training	33.33	−9.22	8
Melanin	15	240	10	0.005	5	31.91		Training	30.56	1.35	3
	16	240	10	0.010	5	51.40		Training	64.91	−13.51	5
	17	240	10	0.050	5	65.97		Test	64.91	1.06	5
	18	240	10	0.100	5	82.77		Training	86.75	−3.98	7
	19	240	10	0.200	5	88.88		Training	86.75	2.13	7
pH	20	240	10	0.010	2	97.89		Test	95.36	2.53	4
	21	240	10	0.010	4	92.97		Training	95.36	−2.40	4
	22	240	10	0.010	5	78.79		Training	64.91	13.88	5
	23	240	10	0.010	7	66.77		Training	64.91	1.86	5
	24	240	10	0.010	9	41.18		Training	41.18	0.00	9
	25	240	10	0.010	11	25.88		Training	24.83	1.04	10
Contact time	1	10	10	0.010	5	9.88		Training	10.05	−0.18	1
	2	20	10	0.010	5	9.33		Training	10.05	−0.72	1
	3	30	10	0.010	5	9.58		Training	10.05	−0.47	1
	4	60	10	0.010	5	9.57		Test	10.05	−0.49	1
	5	120	10	0.010	5	9.54		Test	10.05	−0.51	1
	6	180	10	0.010	5	23.14		Training	23.14	0.00	2
	7	240	10	0.010	5	70.54		Test	64.91	5.63	5
	8	280	10	0.010	5	86.54		Test	89.07	−2.53	6
	9	300	10	0.010	5	90.05		Training	89.07	0.97	6
Initial Cr(VI)	10	240	5	0.010	5	69.61		Training	64.91	4.70	5
	11	240	10	0.010	5	59.12		Training	64.91	−5.79	5
	12	240	50	0.010	5	62.69		Training	64.91	−2.22	5
	13	240	100	0.010	5	49.87		Training	33.33	16.55	8
	14	240	200	0.010	5	25.04		Test	33.33	−8.29	8
Melanin	15	240	10	0.005	5	29.56		Training	30.56	−0.99	3
	16	240	10	0.010	5	53.22		Test	64.91	−11.69	5
	17	240	10	0.050	5	67.97		Training	64.91	3.06	5
	18	240	10	0.100	5	84.65		Training	86.75	−2.10	7
	19	240	10	0.200	5	90.65		Training	86.75	3.90	7
pH	20	240	10	0.010	2	99.84		Training	95.36	4.48	4
	21	240	10	0.010	4	94.76		Training	95.36	−0.60	4
	22	240	10	0.010	5	76.97		Training	64.91	12.06	5
	23	240	10	0.010	7	64.89		Training	64.91	−0.02	5
	24	240	10	0.010	9	39.22		Test	41.18	−1.96	9
	25	240	10	0.010	11	23.77		Training	24.83	−1.07	10

**Table 8:** Model summary structure and statistics of decision tree supervised machine learning algorithm for Cr(VI) removal using fungal biomass, and melanin.

		Fungal Biomass		Melanin	
Model Summary	Total predictor	4		4	
	Important predictor	4		4	
	Terminal node	18		10	
	Minimum terminal node size	1		1	
Statistics		Training	Test	Training	Test
	R^2^, %	96.26	94.60	96.70	95.90
	Root mean squared error	5.0599	5.7665	5.4176	6.1535
	Mean squared error	25.6025	33.2528	29.3509	37.8658
	Mean absolute deviation	2.4584	3.3704	3.5080	3.9997
	Mean absolute percent error	0.0557	0.0832	0.0700	0.0855
	Standard deviation	26.4345	25.3274	30.1164	31.0030
	Number of cases	50	25	50	25

**Table 9:** The expected variables conditions that were projected based on the DT models for fungal biomass, and melanin as well as the expected and actual values of Cr(VI) removal.

DT Model	Investigated Parameters					Cr(VI) Removal, %		Terminal Node
	Contact Time, min	Initial Cr(VI), mg/mL	Fungal Biomass (g)	Melanin (g)	pH	Predicted	Actual	
Fungal biomass	220	70	0.100	-	3.0	99.04	98.28 ± 0.24	6
	230	75	0.050	-	4.0	83.67	86.00 ± 0.56	7
	240	75	0.120	-	5.0	85.33	84.35 ± 0.80	15
Melanin	240	75	-	0.100	4.5	95.36	86.04 ± 0.50	4
	280	70	-	0.070	5.0	89.07	91.22 ± 0.29	6
	220	70	-	0.100	5.0	86.75	80.21 ± 0.28	7

## Data Availability

All data generated or analyzed during this study are included in this published article and the Supplementary Materials.

## Supplementary Materials

The following supporting information can be downloaded at: https://www.mdpi.com/article/10.3390/polym15183754/s1, Table S1. The adsorption of chromium ions on the melanin surface was evidenced by Fourier Transform Infrared Spectroscopy (FT-IR).
