# Supplementary material for: A Comparative Study of Cr(VI) Sorption by Aureobasidium pullulans AKW Biomass and Its Extracellular Melanin: Complementary Modeling with Equilibrium Isotherms, Kinetic Studies, and Decision Tree Modeling
Source: Polymers (Basel). 2023 Sep 14;15(18):3754. doi: 10.3390/polym15183754 (PMC10537747; doi:10.3390/polym15183754)
Supplement: Supplementary file 1 [file polymers-15-03754-s001.zip › polymers-2571508-supplementary.pdf]

A Comparative Study of Cr(VI) Sorption by *Aureobasidium pullulans* AKW Biomass and its Extracellular Melanin: Complementary Modeling with Equilibrium Isotherms, Kinetic Studies, and Decision Tree Modeling

Hala Fakhry [1,*], Abeer A. Ghoniem [2], Fatimah O. Al-Otibi [3], Yosra A. Helmy [4], Mohammed S. El Hersh [2], Khaled M. Elattar [5], WesamEldin I. A. Saber [2,*] and Ashraf Elsayed [6]

Supplementary data

The adsorption of chromium ions on the melanin surface was evidenced by Fourier Transform Infrared Spectroscopy (FT-IR).

Table S1. The representative FT-IR data of fungi biomass (F), chromium adsorbed on fungal biomass (F-Cr), melanin (M), and chromium adsorbed on melanin (M-Cr).

Absorption (cm^{-1})				Appearance	Functional group
F	F-Cr	M	M-Cr		
3272.99	3281.85	3289.57	3311.59	medium	OH or N-H stretching
2921.32	2923.82	2923.12	2922.77	strong, broad	C-H stretching
2851.54	2853.71	2853.50	2852.89	medium	C-H stretching
-	-	2323.61	-	Strong	O=C=O stretching
-	2183.53, 2162.13	2170.03	-	Weak	C-H stretching
-	2152.65, 2085.20	2069.84	-	medium	C-H stretching
-	2050.03	2042.03	-	medium	C-H stretching
-	1987.83	2011.00	-	Weak	C-H stretching
-	-	1740.66	1740.82	Strong	C=O stretching, ester
1608.82	1632.11	1627.26	1624.12	Strong	C=O stretching, amide
1589.78, 1513.92	-		-	medium	N-H bending
1416.81	1443.78	1413.49	-	medium	O-H bending
1363.38, 1330.35	1374.14, 1315.49	1370.51	1374.93	medium	O-H bending
1244.81	1248.68	1232.96, 1201.63	1238.20	medium	C-N stretching
1152.71	1153.77	1149.15	1152.22	Strong	C-O stretching
1027.73	1069.46, 1035.11	1015.69	1025.67	Strong	C-O stretching
840.55	-	-	-	Strong	C-H bending
525.47, 471.52	486.78	482.99, 425.76	-	medium	Phenyl
